# Comparison of Structure and Adsorption Properties of Mesoporous Silica Functionalized with Aminopropyl Groups by the Co-Condensation and the Post Grafting Methods

**DOI:** 10.3390/ma14030628

**Published:** 2021-01-29

**Authors:** Ana-Maria Putz, Mihaela Ciopec, Adina Negrea, Oana Grad, Cătălin Ianăşi, Oleksandr I. Ivankov, Marija Milanović, Ivan Stijepović, László Almásy

**Affiliations:** 1 ”Coriolan Drăgulescu” Institute of Chemistry, Bv. Mihai Viteazul, No.24, 300223 Timisoara, Romania; cianasic@yahoo.com; 2 Faculty of Industrial Chemistry and Environmental Engineering, Politehnica University of Timişoara, Piaţa Victoriei, No.2, Timişoara 300006, Romania; adina.negrea@chim.upt.ro (A.N.); oanagrad88@yahoo.com (O.G.); 3 Frank Laboratory of Neutron Physics, Joint Institute for Nuclear Research, Joliot-Curie 6, 141980 Dubna, Russia; ivankov@jinr.ru; 4 Department of Materials Engineering, Faculty of Technology, University of Novi Sad, Bulevar Cara Lazara 1, 21102 Novi Sad, Serbia; majam@uns.ac.rs (M.M.); ivan.stijepovic@gmail.com (I.S.); 5 Institute for Energy Security and Environmental Safety, Centre for Energy Research, Konkoly-Thege ut 29-33, 1121 Budapest, Hungary

**Keywords:** co-condensation, post-grafting, APTES, chromium adsorption, MCM-41

## Abstract

The adsorptive potential has been evaluated for the aminopropyl functionalized mesoporous silica materials obtained by co-condensation and post grafting methods. Nitrogen sorption, small angle neutron and X-ray scattering (SANS and SAXS) demonstrated high surface area and well-ordered hexagonal pore structure suitable for applications as adsorbents of metals from waste waters. A comparison of Cr(VI) adsorption properties of the materials prepared by different functionalization methods has been performed. The obtained results demonstrated the adsorption capacity due to the affinity of the chromium ions to the amino groups, and showed that co-condensation of tetraethoxysilane (TEOS) and 3-aminopropyl triethoxysilane (APTES) resulted in higher metal sorption capacity of the materials compared to post-synthesis grafting of aminopropyl groups onto the mesoporous silica particles.

## 1. Introduction

Decontamination of toxic pollutants by porous nanomaterials with high removal efficiency became a topic of intense studies in recent years [1]. Among the variety of porous materials, various doped [2] or functionalized silica nanoparticles [3] have been used as adsorbents and it has been established that electrostatic, hydrogen bonding, and hydrophobic interactions are mainly responsible for the adsorption onto the synthesized adsorbents. Mesoporous silica nanoparticles intended for treatment of water pollution as adsorbents, have to possess a high surface area, large pore volume together with a capability to be functionalized with different organic groups [4]. The functionalization of the mesoporous materials has been used as a method of tuning the adsorption properties and is performed by two common ways: the direct co-condensation method (the functional groups are inserted within the silica matrix) [5] and the post-grafting method (the functional groups are attached to the surface of the nanoparticles) [6]. The direct method permits preparation of organic-inorganic hybrid materials in a short time and involves a one-step co-condensation between tetraalkoxy silanes with one or more organoalkoxy silanes through the sol-gel process without or in the presence of a structure directing agent [7]. The advantages of this method are that it gives a more homogeneous distribution of the functional groups but the disadvantage is that it produces materials with a less ordered structure [8,9]. By comparison, the post grafting method uses a two-step procedure: synthesis and functionalization are done at separate stages, thus, it is easier to control particle size, morphology, pore diameter [10], and pore ordering. However, usually it produces nonuniformly distributed organic groups, which can also congregate on the particle external surface blocking the pore entrance [8,11,12]. A recent neutron scattering study with contrast variation showed a more homogeneous surface coverage by SO_3_H groups, when their amount was above a certain threshold [13]. Some interesting differences between the pore morphologies of ordered mesoporous silica prepared by co-condensation and post grafting of organic functional groups have been revealed in previous publications [14,15,16].

Literature studies proved that the functionalization of the silica surface can substantially improve their adsorption capacity particularly for removal of pollutants. Although bare porous silica particles can adsorb and remove certain metal cations due to the presence of surface silanol groups, their metal ion removal or complexation efficiency is limited and the efficiency can be enhanced by grafting functional groups on the surface and inside the pores [17].

In the present study, textural and structural properties of the mesoporous silica with aminopropyl functional groups have been tailored in order to obtain suitable materials for metal ion removal. Synthesis with varying concentrations of APTES have been performed, knowing that this will directly affect the amount of the surface amino groups [18]. For the adsorption study, the Cr(VI) ion has been selected, knowing its toxicity together with its broad industrial use [19]. The presence of chromium as contaminant in the aquatic environment is increased due to the developing industrial activities. The chromium hexavalent cation has a high poisoning level [20,21]. The chromium release in environment occurs via the industrial effluents from tannery, mining, dyes industries, printing, industry of photography, and the drug industries; therefore, the treatment of waste water for chromium removal represents a challenge for environmental protection [22]. Some of the most employed techniques are: electrocoagulation [23], adsorption [24,25], biological treatment [26], and photo catalysis [27].

The adsorptive potential of the synthetized functionalized silica materials has been evaluated by batch adsorption experiments. Preliminary adsorption measurements have been performed for all synthetized samples. Two samples have been selected for more detailed analysis due to their better textural features, higher surface area and pore volumes. The influence of pH, contact time, temperature and the initial concentration on the adsorption capacity of Cr(VI) onto the materials has been evaluated. In order to establish the mechanism of the adsorption, kinetic models and adsorption isotherms have been constructed and analyzed by fitting appropriate models to the experimental data.

## 2. Materials and Methods

### 2.1. Sample Preparation

Reagents were of analytical-reagent grade and used as supplied: tetraethoxysilane (TEOS), (99%, for analysis, Fluka, Steinheim, Germany); 3-aminopropyl triethoxysilane (APTES), (99% Fluka); hexadecyltrimethyl ammonium bromide (CTAB, Sigma-Aldrich, St. Louis, MO, USA); ethanol (Chimopar, Bucuresti, Romania); ammonia solution 25% (Fluka); toluene (Reactivul, Bucuresti, Romania). Silica samples were prepared by using the sol-gel synthesis, using CTAB, TEOS in ethanol and water mixture, and base catalyst NH_3_. The EtOH:H_2_O molar ratio was 1:10. APTES was added in different quantities for each synthesis, while TEOS amount has been kept constant for all the samples.

#### 2.1.1. Synthesis of Functionalized Mesoporous Materials via Co-Condensation Method

One gram of CTAB was added to 385 mL of distilled H_2_O under stirring. After the solution turned clear, 136 mL of ethanol and 46.4 mL of aqueous ammonia solution (25%) was added to the system and it was allowed to mix for 30 min. After that, the silica precursor (7.52 g TEOS + x g of APTES, (x = 0.888 g or 1.410) was poured into the solution slowly under stirring. Stirring was continued for 3 h at room temperature. On the next day, the solid product was recovered by filtration and washed with distilled water, on Whatman filter paper, with repeated filtrations until the pH of the washing water approached the pH value of the distilled water. The extraction of the template has been carried out using 51 mL of acidified ethanol (1 mL of concentrated HCl and 50 mL of ethanol) under magnetic stirring (2 h). Further on, the samples were filtered and washed two times with 20 mL of ethanol, for each washing step. The CTAB extraction procedure has been repeated twice. Next, the material was left for drying at 60 °C, for 24 h. The two samples were named: A-10-co-cond and A-15-co-cond, where the numbers 10 and 15 refer to the percentage of moles of APTES relative to TEOS.

#### 2.1.2. Synthesis of Functionalized Mesoporous Materials via Post-Grafting 

In the post-grafting method, at first mesoporous silica has been prepared and then functionalized, following a modified synthesis recipe from literature [8]. One gram of CTAB was added to 385 mL of distilled H_2_O under stirring. After the solution turned clear, 136 mL of ethanol and 46.4 mL of aqueous ammonia solution (25%) was added to the system and it was allowed to mix for 30 min. After that, 7.52 g of TEOS was poured into the solution slowly under stirring, which continued for 3 h at room temperature. In the next day, the solid product was recovered by filtration and washed several times with distilled water, on Whatman filter paper, with repeated filtrations until the pH of the washing water approached the pH value of the distilled water. The extraction of the template has been carried out by using the same procedure as in the co-condensation synthesis method. Next, the material was left for drying at 60 °C, for 24 h. The ready prepared mesoporous material was grafted with APTES, using the following procedure: the dried mesoporous silica was added to a solution formed from 0.888 g or 1.410 g of APTES and 25 mL of toluene and left for soaking for 24 h, at room temperature. Next, the formed solution was stirred for 6 h. The samples were then filtered and washed with a solution of 25 mL toluene and 25 mL ethanol and left to dry at room temperature for 24 h. The two samples were named: A-10-PG and A-15-PG.

### 2.2. Characterization Methods

FT-IR (Fourier-transform infrared spectroscopy) spectra were recorded on KBr pellets using a JASCO FT/IR-4200 apparatus (SpectraLab, Shimadzu, Japan).

N_2_ adsorption-desorption isotherms were determined by N_2_-physisorption measurements at 77 K using Quantachrome Nova 1200e apparatus (Quantachrome Instruments, Boynton Beach, Florida). Prior to the analysis, the samples were dried and degassed in vacuum at 80 °C for 4 h. The specific surface area was determined by the Brunauer–Emmet–Teller (BET) method in the relative pressure range P/P_0_ from 0.01–0.25. The micropore surface area and external surface area were determined using the de Boer’s V-t method. Pore size distribution was evaluated with Density Functional Theory (DFT) equilibrium model (0.05–1 P/P_0_). The total pore volumes were determined using the point closest to 1 value for the relative pressure P/P_0_.

Small-angle X-ray scattering measurements were performed with an Anton Paar SAXSpace instrument (Anton Paar GmbH, Graz, Austria), equipped with a MYTHEN2 R 1 K one-dimensional detector (Dectris Ltd., Baden-Daettwil, Switzerland) [28,29]. The X-ray generator was operated at 40 kV and 50 mA using Cu K_α_ radiation (λ = 0.1542 nm) and a line source with a Kratky block-collimation system. An exposure time of 30 min was sufficient to give a good signal-to-noise ratio. The scattering intensity I(q) was recorded as a function of the scattering vector q = 4πsinθ/λ, where λ is the wavelength of the incident radiation, and θ is the half of the scattering angle.

Small-angle neutron scattering measurements were performed on the YuMO small-angle spectrometer [30] operating (home-made instrument by FLNP) at the IBR-2 pulsed reactor in Dubna [31]. The scattered neutrons were detected using the time of flight method by a two-detector set-up with ring wire detectors [32]. Measurements were performed on dry powders at room temperature. A vanadium standard was used for the calibration of absolute scattered intensity, while silver behenate sample was used to calibrate distances [33]. The measured scattering curves were corrected for transmission and the background scattering from an empty aluminum sample container using the SAS software [34] (instrument specific software home-made).

Size and morphology of particles were examined using a scanning electron microscope (SEM, JEOL 6460LV, Tokyo, Japan).

Zeta potential of particles was determined by phase analysis light scattering and mixed mode measurement using a Zetasizer Nano ZS with MPT-2 Autotitrator (Malvern Instruments, Malvern, UK). HCl and NaOH of different molarity were used as titrants. The isoelectric point (pH PZC) has been also determined by using the method of bringing the system to equilibrium [35,36]. A 0.1 g for each material was used mixed with 25 mL of KCl solution (0.01 M) at 200 rotations/minute and 298 K temperature, by using a thermostatic water bath with shaking, of Julabo SW23 type. The pH of the KCl solutions were adjusted in the 2–12 range, by using solutions of KOH having concentrations in 0.05–2 M range or by using HCl solutions having concentrations in 0.05–2 M range. The samples were filtered and the pH of the resulting solutions was subsequently determined using a Mettler Toledo, Seven Compact pH meter (Mettler Toledo, GmbH, Greinfensee, Switzerland).

In order to evaluate the adsorptive potential of the synthetized materials, two of the four synthetized samples were selected, the samples A-10-co-cond and A-10-PG, which present the best textural characteristics. The effect of some parameters (contact time, temperature, and the initial concentration) on the adsorption capacity of Cr(VI) on the A-10-co-cond and A-10-PG materials was studied, as well as the effect of the pH, which is linked to the metal ion type in the solution, but also to the surface properties of the sorbents.

The influence of pH was studied by varying the pH value in the 1–6 interval, using the initial concentration of the metallic ion of C_0_ = 100 mg/L, 0.1 g of sorbent, 25 mL metal ion solution, contact time 1 h and temperature 298 K. The Cr(VI) solution of this concentration was obtained by using a stock solution of 1 g/L K_2_CrO_4_ (99%, p.a., ACS, Carl Roth GmbH, Karlsruhe, Germany). The adjustment of the pH was made using different solutions of HNO_3_ and/or NaOH, by varying the concentration in the 0.1–1 M interval.

In order to establish the effect of the contact time and temperature on the materials’ adsorption capacity, 0.1 g of functionalized silica was mixed with 25 mL of Cr(VI) solution with the concentration C_0_ = 100 mg/L. The samples have been shaked for different times (15, 30, 60, 120, and 180 min) using a water bath of Julabo SW23 type at different temperatures (298 K, 308 K, and 318 K) at a rate of 200 rot/min.

In order to evaluate the effect of the initial concentration of Cr(VI) on the materials’ adsorption capacity, solutions of Cr(VI) concentrations of 25, 50, 100, 200, 300, 400, 500, 600, 800, and 1000 mg/L, were used for the sample A-10-co-cond, and concentrations of 5, 10, 25, 50, 75, 100, and 125 mg/L, for the sample A-10-PG. The adsorption study was performed at pH = 3.5, for 2 h and at temperature of 298 K. The residual concentration of Cr(VI) ions was measured using the Varian SpectrAAS 280 FS atomic absorption spectrometer (PTY LTD, Varian, Australia).

The kinetic equations used for the adsorption study are the equation of pseudo-first order (Lagergren model) and the equation of pseudo-second order kinetics (Ho and McKay model) [37]. For the Cr(VI) adsorption on the functionalized mesoporous silica materials, the activation energy *E_a_,* was calculated using the Arrhenius equation and the kinetic constant using the pseudo-second order kinetic model. The Gibbs free energy was calculated using the Gibbs–Helmholtz equation in order to establish if the adsorption process on the material surface is spontaneous. The equilibrium constant (*K_d_*) was calculated as the ratio of the adsorption capacity at equilibrium *q_e_* and the equilibrium concentration *C_e_*. The equilibrium parameters were calculated using the nonlinear expression of the Langmuir isotherm. The experimental data were fitted by nonlinear regression.

## 3. Results and Discussions

### 3.1. FT-IR

**Figure 1:**
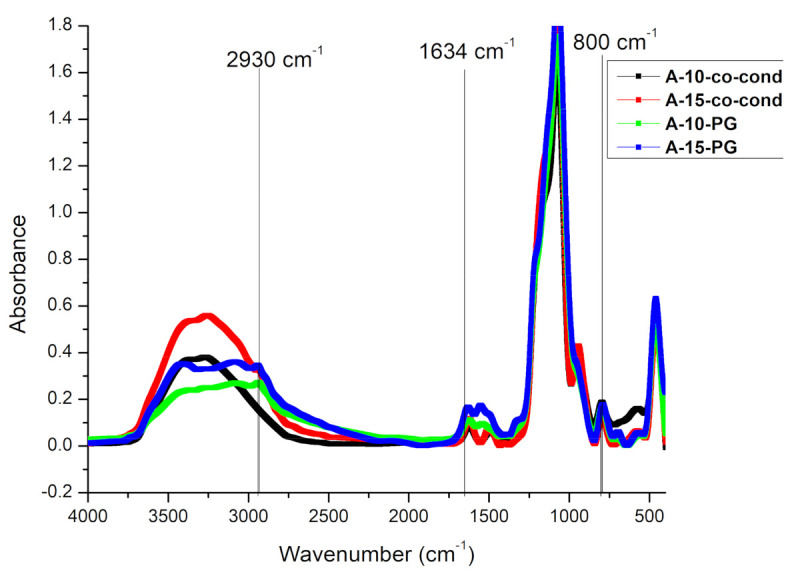
The FT-IR spectra of the functionalized materials.

FT-IR analysis was performed in order to verify if the template removal by extraction was thorough and to check if the material functionalization took place. The FT-IR spectra, shown in Figure 1, reveal the characteristic bands for the aminopropyl functionalized silica materials. All samples show the specific vibration bands assigned to the silica skeleton at 1050, 800, and 450 cm^−1^, corresponding to the asymmetric stretching, symmetric stretching, and bending vibration of the Si-O-Si network, respectively [38]. The presence of the silanol groups were confirmed by the existence of the band centered about 960 cm^−1^, which is associated with the stretching mode of the Si-OH groups [39]. The characteristic vibration bands of the surfactant molecules at 2920 cm^−1^ and 2850 cm^−1^ were observed in the samples before the CTAB extraction (data not shown), while after extraction by acidified ethanol solutions these bands are not present [39]. The small peak at 2930 cm^−1^, for the functionalized materials corresponds to the methylene vibrations in the aminopropyl groups. Additionally, two peaks confirm the presence of amino groups, one at 1634 cm^−1^, which is typical for protonated amino groups and another one at 800 cm^−1^ for the torsion-type vibration of amino groups [40], which is not present in the non-functionalized material. Therefore, the FT-IR data confirmed the presence of amino groups in the functionalized materials.

### 3.2. Nitrogen Adsorption Measurements

**Figure 2:**
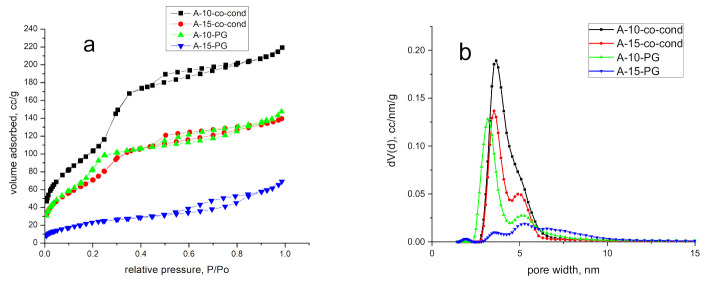
N_2_ adsorption-desorption isotherms (**a**) and pore size distributions obtained by DFT (**b**) method of the functionalized mesoporous silica samples.

Figure 2 comparatively presents the N_2_ adsorption-desorption isotherms. The isotherms are of Type IV(a) following the IUPAC classification [41]. The presence of the hysteresis indicates that pore condensation takes place and for all samples the hysteresis loops are closing at 0.42 P/P_0_. A H2 (b) type hysteresis has been observed for all samples. This type of hysteresis is encountered for samples where pore blocking takes place and pores have the shape of ink-bottle with larger neck. In Figure 2b, the pore size distributions obtained by DFT method are shown. The sample obtained by co-condensation, A-10-co-cond, presents a unimodal size distribution with pores around 4 nm in diameter, whereas in the case of post-grafting (A-10-PG) the distribution becomes bimodal with the majority of pores around 3.5 nm and a lower percentage of 6 nm pores. In case of the sample A-15-co-cond, prepared with higher amount of APTES, a bimodal distribution with pores similar to A-10-PG was observed. For the post grafting case, in the A-15-PG sample a broad multimodal distribution is observed with pores between 3 and 10 nm.

The textural parameters are collected in Table 1. By analyzing the data, it was observed that the samples with lower quantity of APTES presented higher surface area and higher volume of pores. For the post-grafting synthetized samples, we observed a drastic surface area decrease, especially in case of samples with higher quantity of APTES. The pore size in the A-15-PG sample, as calculated by the DFT method was above 5 nm, which is unrealistic for the MCM-41 material templated with CTAB. This result shows that the assumption of cylindrical pore shape is no more valid, and the sample porosity is heterogeneous due to the high amount of APTES. The highest total pore volume and surface area was obtained for sample A-10-co-cond. The different textures of the materials are observed also in the surface fractal dimensions evaluated from Frenkel–Halsey–Hill (FHH) method. The FHH method is used to determine the fractal geometry and calculate their surface irregularities and porous structure [42]. When the value of Df is 2, the material presents a surface fractal and if the value of Df is 3, the material presents a mass fractal. For all analyzed samples, a mass fractal behavior was observed, from the FHH data of Table 1 by accounting for Adsorbate Surface Tension Effects.

### 3.3. Small angle X-ray Scattering (SAXS) and Small Angle Neutron Scattering (SANS) Analysis of the Particle Morphology and Pore Structure

**Figure 3:**
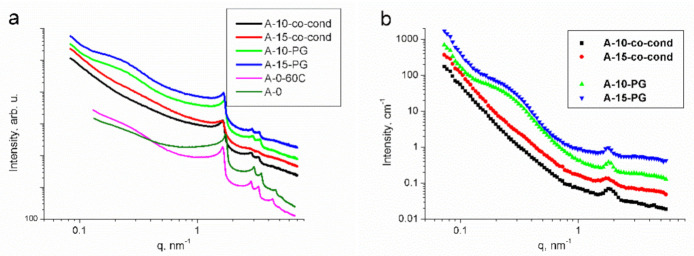
Small angle X-ray scattering (SAXS) (**a**) and Small Angle Neutron Scattering (SANS) (**b**) scattering curves of the parent and the functionalized mesoporous silica samples. The data have been rescaled for better visibility. A-0 refers to the calcined sample prepared only with tetraethoxysilane (TEOS), and A-0-60C is the same material prior to calcination.

The SANS and SAXS patterns of a given material can, in general, be different, due to the different contrasts between the constituent phases, which depend on the type of radiation. In our case, the system consists of two dominant phases, the pores and the silica matrix [43], and only a small amount of aminopropyl groups situated on the interfaces. Therefore, the two radiations see essentially the same structure, apart from the difference in the absolute intensities [44]. The SAXS scattering curves of the four samples are shown in Figure 3a, together with the scattering curves of the as-prepared and the calcined samples prior to functionalization.

The most prominent features are the diffraction peaks indicating the 2-dimensional hexagonal pore structure, characteristic to MCM-41 materials. The 100 peak is strong for all samples, and the 110 and 200 peaks are rather weak in the A-15-co-cond sample. This shows that the increase of the amount of organic silica precursor in the co-condensation leads to the decrease of the long-range ordering of the parallel channels. Such decrease of the ordered porosity has been found for composite MCM-41 prepared using a mixture of TEOS and methyl-triethoxysilane precursors, both in acidic and alkaline conditions [38,45,46,47]. Calcination at 550 °C caused a shrinkage of the unit cell dimension by 6%, as commonly observed in ordered mesoporous silica prepared with CTAB [43,45]. The SANS patterns (Figure 3b) show the same behavior, with the difference that the higher order peaks are not visible due to the lower angular resolution of this method. The lowest ordering of the A-15-co-cond sample is seen from the widening of the main 100 diffraction peak. A marked difference between the samples made by co-condensation and post grafting is the broad hump that appears on both SANS and SAXS patterns of the post-grafted samples, which originates from the uneven distribution of the aminopropyl moieties. Their characteristic cluster size *D* can be deduced from the position of the hump q*, calculated as *D* = 2π/q*, giving a value of 20–30 nm. This feature is absent in the scattering curves for the samples prepared by co-condensation, pointing to the homogeneous distribution of the aminopropyl groups within the mesoporous spherical silica particles.

### 3.4. SEM Analysis

**Figure 4:**
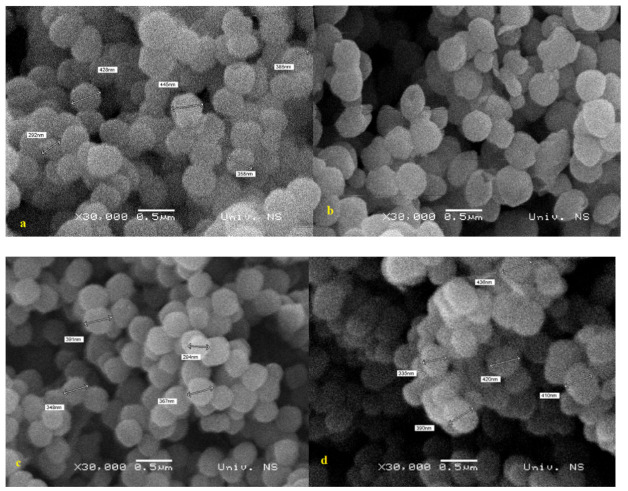
SEM images of the functionalized mesoporous silica particles: A-10-co-cond (**a**), A-15-co-cond (**b**), A-10-PG (**c**), and A-15-PG (**d**) samples.

Morphology of the samples is presented in Figure 4. All samples consist of spherical silica particles with narrow size distribution with particle size of 0.3–0.5 μm, characteristic for the Stöber synthesis in alkaline conditions. At the magnification provided by the SEM, there were no substantial differences observed in particle sizes or shapes for the four samples prepared by co-condensation and post grafting.

### 3.5. Sorption Studies

#### 3.5.1. Zeta Potential Measurements

**Figure 5:**
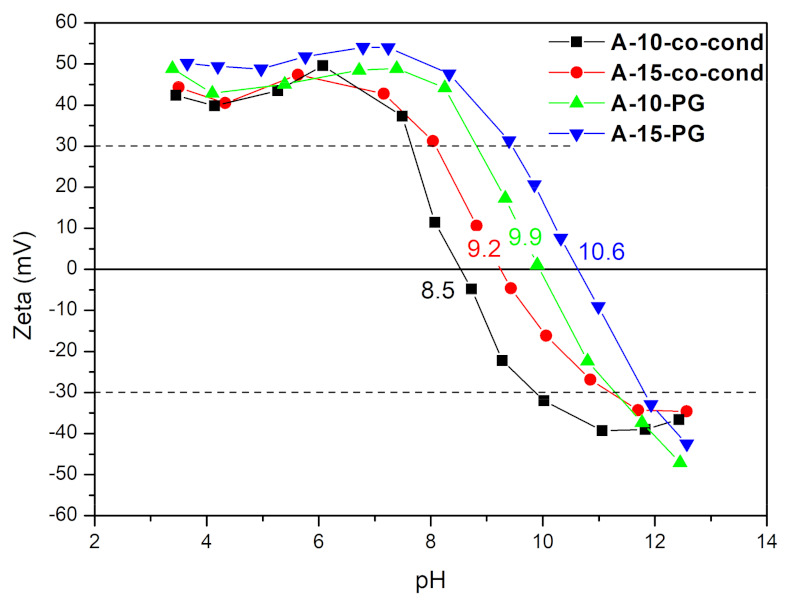
Zeta potential of the samples measured as a function of pH.

The colloidal stability of functionalized mesoporous silica dispersed in aqueous media was investigated by measuring the zeta potential and results are shown in Figure 5. Since zeta potential is related to the surface charge on the particles, its value indicates the degree of electrostatic or steric repulsion between the particles. It is generally accepted that the particles are electrostatically stable if an absolute value of the zeta potential is higher than 25–30 mV [48]. In that manner, from the results shown in Figure 5, it can be seen that the particles are stable and positively charged in a wide range of pH. The stability increases in the samples with higher amount of APTES, implying that functionalization with APTES successfully neutralized the negative hydroxyl ions and deposited more positive amino ions on the surface of the silica to become positively charged. This way, particle surface becomes suitable for absorption of the negatively charged HCrO_4_^−^, CrO_4_^2−^, or Cr_2_O_7_^2−^ anion groups. In addition, the post grafting method further extends the colloidal stability of the silica particles dispersions. The isoelectric point (pI), where particles carry no net electric charge on their surfaces, is in the range from 8.5 to 10.6, as presented in Figure 5.

#### 3.5.2. Point of Zero Charge (pZc) Determined by Using the Method of Bringing to Equilibrium

**Figure 6:**
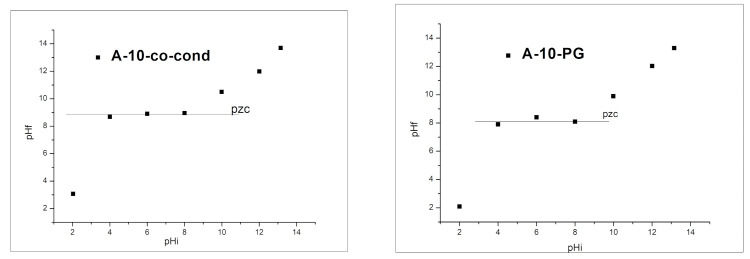
Point of zero charge, pZc of the A-10-co-cond (**left**) and A-10-PG (**right**) samples.

For exploitation of materials for adsorption purposes, it is very important to know their acidic-basic properties. In order to measure the point of zero charge using the method of bringing the system to equilibrium, pH_pZc_ associated with each material, the final value of the pH (pH_f_) has been graphically represented as a function of the initial value of pH, pH_i_ (Figure 6).

In the studied interval of pH_i_ of 2–10, the materials have buffering capacities. The A-10-co-cond material has the value for pZc = 8.5, within the interval pH = 4–8, and the material A-10-PG has pZc = 8, in the interval pH = 4–8; showing the pH range of possible usage of these materials for adsorption. The NH_2_ groups which appear due to the functionalization and also the OH groups from the silica surface, are all protonated at low pH values, which leads to a surface positively charged at pH ~ 3.

#### 3.5.3. Batch Adsorption Studies

##### Effect of pH

**Figure 7:**
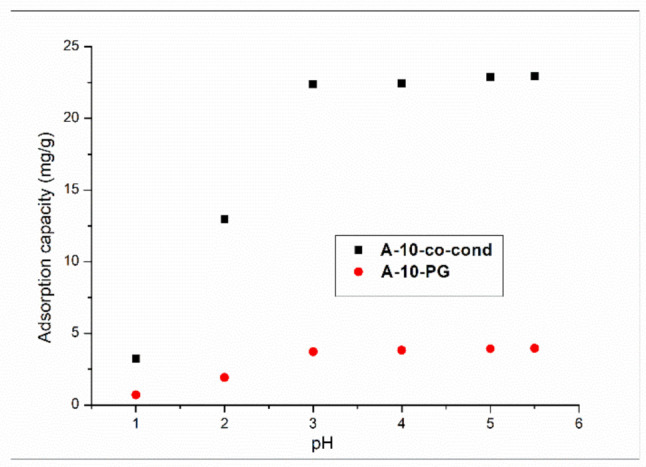
The pH dependence of the adsorption capacity the A-10-co-cond and A-10-PG samples.

For the pH influence on the adsorption process of Cr(VI), literature studies show that the precipitation of Cr(OH)_3_ starts at pH = 5.86 [49,50]. The pH effect on the adsorption capacity of Cr(VI) for both materials is presented in Figure 7.

For both materials the adsorption capacity of the chromium ions is increasing in the pH = 1–3 interval. At the pH values pH > 3, the adsorption capacity remains constant. Moreover, it has been observed that the optimum pH for the Cr(VI) ions is pH~3. The maximum adsorption capacity for both materials is in the pH = 3–5 interval; above this value the chromium will precipitate. In an aqueous solution with pH value in between 3–5, the predominant and equilibrium chromium species are HCrO_4_^−^ and Cr_2_O_7_^2−^. With the increase of the pH value, the CrO_4_^2−^ appears. We may say that a CrO_4_^2−^ ion needs two active centers of adsorption, and the HCrO_4_^−^ needs one single center of adsorption. Therefore, we may observe an increase of the adsorption of Cr(VI) due to the formation of HCrO_4_^−^ in high quantity at pH ~ 3. The positively charged functional groups present in the synthetized materials, may be electrostatically bonded or by hydrogen bonds, to the anions HCrO_4_^−^, CrO_4_^2−^, or Cr_2_O_7_^2−^, in function of the pH. With the increase of pH, the protonation is decreasing, and in alkaline conditions the OH^-^ ions are linked to Cr(VI) ions, resulting, therefore, in a decreasing of the adsorption capacity [51,52,53].

##### Contact Time and Temperature Effect. Kinetic and Thermodynamic Studies

**Figure 8:**
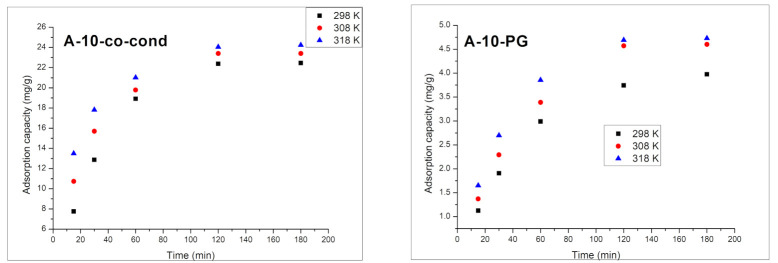
Adsorption capacity dependence on the contact time and temperature for the A-10-co-cond (**left**) and A-10-PG (**right**) samples.

In Figure 8, the effects of the contact time and temperature on the adsorption process of Cr(VI) on the A-10-co-cond and the A-10-PG functionalized silica materials are presented.

**Figure 9:**
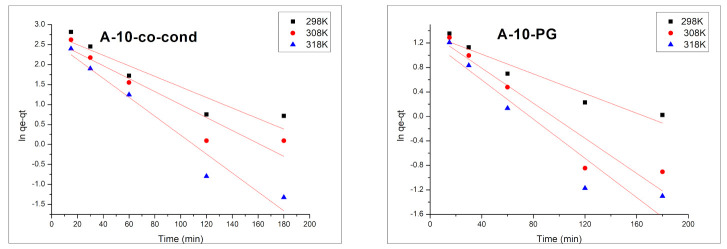
Pseudo-fist order kinetic model for the A-10-co-cond (**left**) and A-10-PG (**right**) samples.

**Figure 10:**
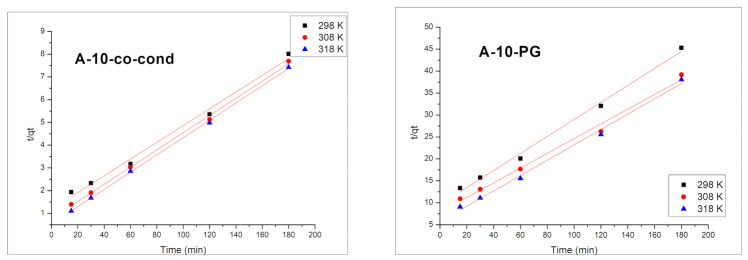
Pseudo-second order kinetic model for the A-10-co-cond (**left**) and A-10-PG (**right**) samples.

With the increase of the contact time, the adsorption capacity for Cr(VI) is increasing, reaching the maximum value around 120 min. After this time, the adsorption capacity remains approximately constant. The adsorption process of Cr(VI) is weakly influenced by temperature. In order to reveal the mechanism of Cr(VI) removal by adsorption, the experimental data were modelled using the kinetic models of pseudo-first order (Figure 9) and pseudo-second order (Figure 10), and the kinetics data are presented in Table 2.

Kinetics information regarding Cr(VI) adsorption onto adsorbent materials were obtained by modelling the experimental data using Lagergren pseudo-first order model and Ho and McKay pseudo-second order model.

Integrated form of the pseudo-first order kinetic model is expressed by Equation (1).

ln (*q_e_* − *q_t_*) = ln*q_e_* − *k*_1_*t* (1)

where *q_t_* and *q_e_* represent the adsorption capacities at time *t* and at equilibrium time, respectively (mg/g), and *k*_1_ is the specific adsorption rate constant (min^−1^).

Linear form of the pseudo-second order rate expression is given by Equation (2):

(2) $$\frac{t}{q_{t}}=\frac{1}{k_{2}q_{e}^{2}}+\frac{t}{q_{e}}$$

where *k*_2_ is the pseudo-second order constant (min^−1^(mg/g)^−1^)

The validation of these two kinetic models is done by the value of the regression coefficient *R*^2^ and by comparing the calculated and measured adsorption capacity *q_e,calc_*. From the data presented in Table 2 it is observed that the kinetic model of pseudo-second order is the in describing the adsorption process. Moreover, the obtained theoretical values of the adsorption capacities, *q_e,calc_* are close to the obtained experimental values, *q_e,exp_*.

The adsorption of Cr(VI) ions on the materials’ surface took place in one step because the second order kinetics analysis implies that the rate controlling step is the elementary reaction between the adsorbate and the adsorbent [54,55,56].

**Figure 11:**
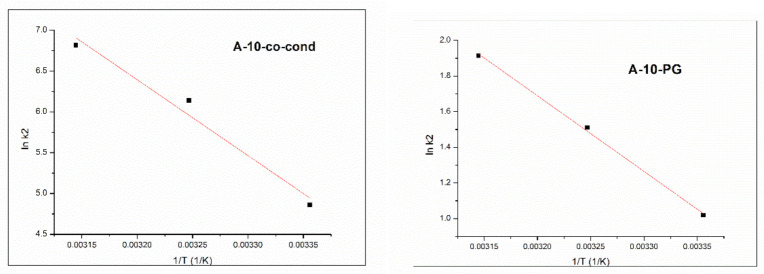
Arrhenius plots for adsorption process for the A-10-co-cond (**left**) and A-10-PG (**right**) samples.

The activation energy value, *E_a_*, may give information about the nature of the process, if it is physical or chemical. *E_a_* was calculated by using Arrhenius equation (Figure 11).

It may be observed that energy of activation is *E_a_* < 40 kJ/mol (28.4 kJ/mol for the A-10-co-cond and 35.2 kJ/mol for the A-10-PG sample), which means that the adsorption process of Cr(VI) on these two materials is of physical nature [57]. The temperature effect on the removal process of Cr(VI) by adsorption has been studied at 3 different temperatures, 298 K, 303 K and 318 K. The adsorption process is stimulated by temperature increase. The increase of the adsorption capacity of the material at increasing temperatures may be due to the activation of the adsorbent surface.

The values for Δ*H*° and Δ*S*° were calculated by linearization of the Van’t Hoff equation. Based on the obtained experimental data the values of thermodynamic parameters were evaluated: free Gibbs energy (Δ*G*^0^), free enthalpy (Δ*H*^0^), and free entropy (Δ*S*^0^) were calculated by using relations:

Δ*G*^0^ = −*RT*ln*K_d_* (3)

where:

(4) $$K_{d}\;=\;\frac{C_{\mathrm{Ae}}}{C_{e}}$$

and

(5) $$\log\;K_{d}\;=\;\frac{\Delta S^{0}}{2.3\;R}-\frac{\Delta H^{0}}{2.303\;\mathrm{RT}}$$

where: *R* is the gas constant, *K_d_* is the equilibrium constant, *T* is the temperature (K), *C_Ae_* is the equilibrium concentration of Cr(VI) on adsorbent (mg/L), and *C_e_* is the equilibrium concentration of Cr(VI) in the solution (mg/L).

**Figure 12:**
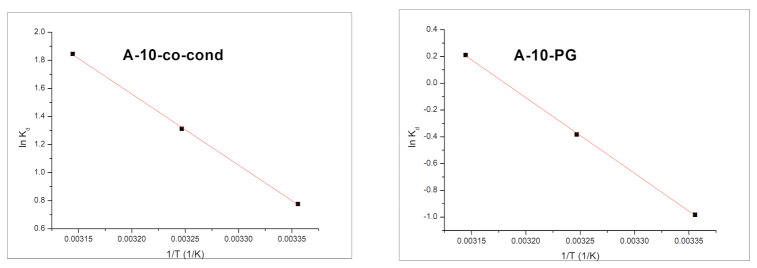
The ln*K_d_* versus 1/*T* plots for the A-10-co-cond (**left**) and A-10-PG (**right**) samples.

Enthalpy and entropy associated with the studied adsorption process are evaluated from the slope and the intercept of linear dependence of ln*K_d_* vs. 1/*T* (Figure 12). Based on these values the free Gibbs energy can be calculated. Values of thermodynamic parameters obtained for Cr(VI) adsorption on adsorbent materials are presented in Table 3.

The values for Δ*H*^0^ and Δ*S*^0^ were calculated by linearization of the Van’t Hoff equation (Figure 12), and the thermodynamic parameters are shown in Table 3.

The negative values of Gibbs free energy (Δ*G*^0^) confirm the process feasibility and the spontaneous nature of Cr(VI) ion adsorption by the two materials. Once the temperature increases from 298 K to 318 K, the values of Δ*G*^0^ become more negative, suggesting that the adsorption process is favored at higher temperatures. The positive values obtained for Δ*H*^0^ and Δ*S*^0^ confirm that the adsorption process is endothermic and that the entropy increases at the interface of the two materials with the attachment of the Cr(VI) ions.

##### Adsorption Isotherms

**Figure 13:**
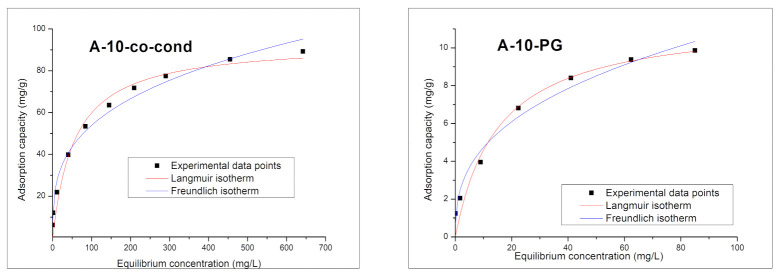
Adsorption isotherms for the A-10-co-cond (**left**) and A-10-PG (**right**) samples.

In order to describe the mechanism of adsorption process of Cr(VI) onto the A-10-co-cond and A-10-PG materials, the Freundlich and Langmuir models were used. The experimental data and the model fits are presented in Figure 13, and the specific parameters for the studied isotherms in Table 4.

The highest value for the regression coefficient, *R*^2^, was obtained using the Langmuir (~0.99) model, therefore, we may conclude that the removal process of Cr(VI) by adsorption onto the two materials is better described by the Langmuir model. The A-10-co-cond material has the maximum adsorption capacity of 93.6 mg/g, obtained by fitting the experimental data using the Langmuir model while for the A-10-PG the fitted adsorption capacity is 11.5 mg/g. The maximum adsorption capacity experimentally determined is 85.4 mg/g for A-10-co-cond sample and 9.4 mg/g for the A-10-PG sample, very close to the theoretical values determined by Langmuir model, proving that this model describes the best the adsorption process. The A-10-co-cond has higher affinity for Cr(VI) ions compared to the A-10-PG material.

### 3.6. Comparison with Other Adsorbents for Cr(VI) Removal

Some examples of others materials utilized for Cr(VI) ions removal are presented in Table 5. 

The comparison of the performance of various materials shows that the amino modified mesoporous silica prepared by co-condensation in the present study is superior in Cr(VI) removal to the most of the materials listed in the cited sources, with the exception of some organic polymer conjugated silica nanoparticles, such as poly(catechol-tetraethylenepentamine)@SiO_2_-NH_2_ [66], polyethylenimine-silica nanocomposite [69], or polypyrrole-silica [70]. Our study shows that the simple method of co-condensation of functionalized silica precursors in the presence of pore forming surfactant molecules leads to cheap and competitive materials suitable for metal ion removal from waste waters.

## 4. Conclusions

The morpho-structural and adsorption properties of mesoporous silica functionalized with APTES by the co-condensation and the post grafting methods were evaluated. From the performed studies it was found that adsorption capacity of Cr(VI) was almost ten times higher for the material prepared by co-condensation compared to the one prepared by post-grafting (*q_exp_* = 85.5mg/g vs. *q_exp_* = 9.4mg/g).

The influence of some parameters (pH, contact time, temperature, and the initial concentration) on the adsorption capacity of Cr(VI) of both materials was studied. The results demonstrated that the adsorption process is dependent on: pH, the contact time between the adsorbent/chromium solution, temperature, and on the initial concentration of Cr(VI). In order to establish the mechanism of the adsorption process, the experimental data were analyzed by using different kinetic models.

Information regarding the adsorption kinetics was obtained by fitting the experimental data with pseudo-first order and pseudo-second order kinetic models. Obtained data are better described by the pseudo-second order kinetic model. Further, the adsorption data were modelled using Langmuir and Freundlich adsorption isotherms. The modelling of the obtained experimental data showed that the Langmuir isotherm best describes the adsorption process, because the correlation coefficient *R*^2^ approaches 1 and the maximum calculated adsorption capacities (93.6 mg/g for A-10-co-cond and 11.5 mg/g for A-10-PG) are close to the experimentally determined values.

The thermodynamic studies showed that the adsorption process is spontaneous, endothermic, and temperature dependent. The sample obtained by co-condensation had an increased affinity for Cr(VI) ions compared to the post-grafted material, which may be explained by the increased specific surface area and pore volume of this material, and the possibly higher amount of surface amine groups in the pore channels due to their even distribution provided by the co-condensation method. SAXS and SANS data show that the increase of the amount of organic silica precursor in the co-condensation process leads to the decrease of the long-range order of the parallel channels. The FHH analysis showed that the rugosity of materials was higher for samples with lower quantity of APTES. The colloidal stability increased in the samples with higher amount of APTES, implying that functionalization with APTES leads to the positively charged particle surface, making it suitable for absorption of the of negatively charged HCrO_4_^−^, CrO_4_^2−^, or Cr_2_O_7_^2−^ anion groups. In addition, the post grafting method leads to higher colloidal stability of the silica particles. The isoelectric point (pI) for all synthetized samples was in the range from 8.5 up to 10.6.

## Figures and Tables

**Table 1:** Textural parameters of the samples determined by N_2_ sorption.

Sample	S_BET_ (Specific Surface Area (Total)) (m^2^/g)	Micropore Area (m^2^/g)	d_DFT_ (nm)	VT (Total Pore Volume) (cm^3^/g)	df _FHH (ads/des)_
A-10-co-cond	500	183	3.6	0.34	2.13/2.71
A-15-co-cond	323	145	3.5	0.22	2.18/2.72
A-10-PG	416	289	3.2	0.23	2.18/2.72
A-15-PG	93	37	5.3	0.11	1.90/2.63

**Table 2:** Kinetic parameters for Cr(VI) adsorption onto the A-10-co-cond and A-10-PG materials.

*T*, K	*q*_*e*,*exp*_, mg/g	Pseudo-First Order Kinetic Model			Pseudo-Second Order Kinetic Model		
		*q*_*e*,*calc*_, mg/g	*k*_1, _min^−1^	R^2^	*q*_*e*,*calc*_, mg/g	*k*_2, _min^−^^1^(mg/g)^−^^1^	R^2^
**A-10-co-cond**							
298	22.4	15.8	0.0132	0.8849	26.3	128.9	0.9994
308	23.4	13.7	0.0162	0.8975	26.4	625.6	0.9982
318	24.3	13.5	0.0237	0.9588	27.1	910.6	0.9926
**A-10-PG**							
298	3.7	1.2	0.008	0.9355	5.7	2.77	0.9974
308	4.5	1.8	0.013	0.9145	5.8	4.33	0.9913
318	4.7	2.2	0.016	0.918	5.2	6.78	0.9998

**Table 3:** Thermodynamic parameters for the adsorption of Cr(VI) onto materials.

Materials	Temperture, K	ΔG^0^, kJ/mol	ΔH^0^, kJ/mol	ΔS^0^, kJ/mol∙K
A-10-co-cond	298	−1.9	42.1	0.14
	308	−3.4		
	318	−4.8		
A-10-PG	298	−1.6	42.9	0.15
	308	−3.1		
	318	−4.6		

**Table 4:** Parameters of the isotherm models for adsorption of Cr(VI) on the adsorbent materials.

Freundlich Isotherm			Langmuir Isotherm		
*K_F_*, mg/g	1/*n*	R^2^	*K_L_*, L/mg	*q_m calc_*, mg/g	R^2^
**A-10-co-cond**					
13.2	0.31	0.9848	0.02	93.6	0.9969
**A-10-PG**					
2.01	0.36	0.9749	0.06	11.5	0.9949

**Table 5:** Adsorption capacities of some adsorbents used for Cr(VI) ions removal, their morphologies, specific surface area, and the total pore volumes, cited in recent literature.

Adsorbent and Morphology	Adsorption Capacity, mg/g	Specific Surface Area (S_BET_) and Total Pore Volume (V_T_)	Reference
Orange peel	7.14	-	[58]
Potato peel	3.28	-	[59]
Banana peel	3.35	-	[60]
Pea pod	4.33	-	[61]
Litchi peel	7.05	-	[62]
Coconut shell	8.73	S_BET_ = 1.49 m^2^/g; V_T_ = 0.683 cm^3^/g	[63]
Garlic peel	9.22	S_BET_ = 3.21 m^2^/g; V_T_ = 0.191 cm^3^/g	[63]
Amorphous silica nanoparticle	0.4	S_BET_ = 387 m^2^/g; V_T_ = 0.66 cm^3^/g	[64]
Ordered mesoporous silica nanoparticle	1.3	S_BET_ = 792.1 m^2^/g; V_T_ = 2.11 cm^3^/g	[64]
amino-functionalized amorphous silica nanoparticle	33.9	S_BET_ = 189.9 m^2^/g; V_T_ = 0.60 cm^3^/g	[64]
Amino-functionalized mesoporous silica nanoparticle	42.1	S_BET_ = 213.5 m^2^/g; V_T_ = 1.42 cm^3^/g	[64]
Amino functionalized mesoporous silica (hexagonal pore structure)	50	S_BET_ = 721 m^2^/g;	[65]
Poly(catechol-tetraethylene-pent-amine) @SiO_2_-NH_2_	400.8	-	[66]
Aminopropyl functionalized mesoporous silica by post-grafting (Primary particles of 50–60 nm in size formed agglomerates of 700–800 nm in size)	39.3;82.5; 84.3	S_BET_ = 1379.6 m^2^/g; V_T_ = 1.7 cm^3^/g S_BET_ = 857.88 m^2^/g; V_T_ = 0.0698 cm^3^/g S_BET_ = 682.06 m^2^/g; V_T_ = 0.256 cm^3^/g	[67]
Amine functionalized mesoporous silica with well-ordered hexagonal pore structure	47.27	-	[68]
Polyethylenimine-silica nanocomposite Particles with near spherical and elliptical morphologies, with size in range 200–300 nm, uniformly distributed Si and N atoms	183.7	S_BET_ = 21.6 m^2^/g; V_T_ = 0.07 cm^3^/g	[69]
Polypyrrole/hollow mesoporous silica particle (energy-dispersive X-ray spectroscopy analysis showed the uniform distribution of N element of pyrrole)	382	-	[70]
A-10-co-cond	85.4	S_BET_ = 500 m^2^/g; V_T_ = 0.34 cm^3^/g	This paper
A-10-PG	9.4	S_BET_ = 416 m^2^/g; V_T_ = 0.23 cm^3^/g	This paper

## Data Availability

The data presented in this study are available on request from the corresponding author.
